# Neutropenic Tonsillar Ulcer as the Initial Presentation of Acute Myeloid Leukemia in a Young Woman: A Case Report

**DOI:** 10.7759/cureus.104818

**Published:** 2026-03-07

**Authors:** Mustafa Al Hassani, Aqeel Saleem, Zaid Al Hassani, Ali Al Hassani, Mohammed F Farooqi

**Affiliations:** 1 Internal Medicine, Sheikh Tahnoon bin Mohammed Medical City, Al Ain, ARE; 2 Infectious Disease, Sheikh Tahnoon bin Mohammed Medical City, Al Ain, ARE; 3 Faculty of Medicine and Health Science, United Arab Emirates University, Al Ain, ARE; 4 General Practice, Sheikh Tahnoon bin Mohammed Medical City, Al Ain, ARE; 5 Faculty of Medicine and Health Sciences, United Arab Emirates University, Al Ain, ARE

**Keywords:** acute myeloid leukemia, case report, neutropenic ulcer, oral ulceration, young adult

## Abstract

Neutropenic ulceration can represent the initial presentation of acute myeloid leukemia (AML), but diagnostic delay may occur when recurrent oral ulcers in young adults are attributed to benign causes. We report a case that highlights the diagnostic challenges posed by neutropenic oral ulceration as the first manifestation of AML. A 25-year-old woman with recurrent oral ulcers presented with severe throat pain persisting for one month despite prior cauterization and antiviral therapy. Examination revealed a large tonsillar ulcer measuring approximately 4 × 4 cm. Initial investigations demonstrated severe neutropenia (absolute neutrophil count 0.27 × 10⁹/L) with evolving pancytopenia on serial testing, with a background of mild neutropenia documented months before presentation. Initial peripheral blood flow cytometry was non-diagnostic, showing granulocytopenia with relative monocytosis and no definitive clonal features. The patient developed febrile neutropenia and was treated with broad-spectrum antibiotics and supportive care. Given persistent severe neutropenia with progressive pancytopenia despite supportive management and a non-diagnostic initial flow cytometry, repeat flow cytometry four weeks later detected circulating myeloid blasts (7.7%) with an abnormal immunophenotype. Bone marrow examination confirmed AML with monocytic differentiation, demonstrating markedly hypercellular marrow with >70% blasts and occasional Auer rods. Oral manifestations of AML, including ulceration, mucosal bleeding, and gingival involvement, may be presenting features, particularly in monocytic subtypes, and neutropenic ulceration may rarely be the dominant initial manifestation in young adults. This case underscores the importance of comprehensive hematologic screening in patients with persistent oral ulcers, particularly when accompanied by progressive neutropenia or refractory ulceration. Neutropenic ulceration should prompt systematic evaluation for underlying bone marrow disorders. Serial hematologic monitoring, with repeat flow cytometry and/or bone marrow evaluation, is warranted when initial studies are non-diagnostic. Early recognition and structured diagnostic pathways may reduce diagnostic delay and improve outcomes.

## Introduction

Recurrent oral ulceration is a common presenting complaint, occurring most frequently in adolescents and young adults, and is most often attributable to benign recurrent aphthous stomatitis (RAS) [1-3]. Although many cases are self-limited, persistent, atypical, or severe ulcers should prompt evaluation for systemic disease because the differential diagnosis is broad and includes inflammatory disorders (e.g., Behçet disease and inflammatory bowel disease), hematologic disorders, infections, nutritional deficiencies, drug-induced ulceration, and malignancy [2,4].

Hematologic disorders are an important and potentially life-threatening cause of oral ulceration and may be overlooked during initial assessment [5]. Hematinic deficiencies (iron, folate, and vitamin B12) are significantly associated with recurrent aphthous ulceration, supporting routine screening with complete blood count and hematinic assays in patients with recurrent or persistent ulcers [6]. Oral findings such as ulceration, gingival bleeding, petechiae, and mucosal pallor may represent the first sign of an underlying hematologic malignancy, including acute leukemia [7]. Neutropenic ulceration is a distinct clinicopathologic entity in which mucosal ulcerations occur in the setting of primary or secondary neutropenia, with neutropenia considered the principal causative factor [8]. Pathophysiology involves impaired mucosal barrier homeostasis due to reduced oral neutrophil counts, altered neutrophil trafficking into tissues, and defective neutrophil function, predisposing to ulceration and periodontal disease [9]. Expert consensus guidance for difficult or complicated oral ulcers recommends escalation for lesions persisting beyond approximately two weeks or failing empiric therapy, including hematologic screening and biopsy when appropriate, and urgent hematology referral when unexplained cytopenias or abnormal cells are present [10].

Acute myeloid leukemia (AML) is the most common acute leukemia in adults, with an annual incidence of approximately three to four per 100,000 persons and a median age at diagnosis in the seventh decade of life [11,12]. Oral manifestations can be an early or presenting feature of AML, particularly in monocytic subtypes, through a combination of marrow failure-related cytopenias and leukemic tissue involvement [7]. We report the case of a 25-year-old woman with AML with monocytic differentiation who presented with a large, persistent tonsillar ulcer as the dominant initial manifestation, without typical constitutional symptoms at presentation. Inflammatory etiologies such as IBD were considered, given her chronic abdominal symptoms and a reportedly elevated fecal calprotectin; however, prior endoscopic evaluation with biopsies was reportedly normal, and a definitive IBD diagnosis had not been established, while progressive neutropenia and evolving cytopenias ultimately prompted serial hematologic reassessment leading to diagnosis [4].

## Case presentation

A 25-year-old woman with morbid obesity (body mass index 43 kg/m²), prediabetes, and dyslipidemia presented to the emergency department (Day 1) with severe mouth and throat pain persisting for one month. Medication history before admission was notable for symptomatic analgesics only, including paracetamol and non-steroidal anti-inflammatory drugs, and a recent course of acyclovir prescribed at an external facility for presumed herpetic ulceration. No other regular medications or recent drug exposures associated with neutropenia were identified from the available history. She reported multiple painful oral ulcers with progressive odynophagia and dysphagia, which markedly reduced oral intake and were associated with unintentional weight loss.

On initial assessment, she was afebrile and hemodynamically stable. Approximately one month before presentation, she had been evaluated at an external facility where cauterization was performed for presumed herpetic ulceration. Despite this intervention and courses of acyclovir, the lesion did not heal and remained significantly painful. She denied hoarseness, dyspnea, fever, night sweats, easy bruising, urinary symptoms, or eye pain at presentation.

She recalled similar episodes of oral ulceration earlier in the year (approximately eight months and three to four months before admission), both resolving spontaneously within one week. She also reported an eight-year history of chronic abdominal pain with alternating diarrhea and constipation, typically worsening around menses. Prior gastrointestinal evaluation included an esophagogastroduodenoscopy (approximately 2.5 years earlier) showing mild non-erosive gastritis with negative *Helicobacter pylori *testing, and repeat esophagogastroduodenoscopy and colonoscopy with biopsies approximately three months before admission that were reportedly normal. Fecal calprotectin was reportedly markedly elevated (in the 700s), and magnetic resonance enterography was planned but not completed due to missed follow-up. Rheumatology assessment approximately two weeks before admission found no evidence of systemic autoimmune disease, with a negative workup for systemic lupus erythematosus and rheumatoid arthritis.

On examination, she was alert and oriented but appeared uncomfortable due to pain. Oropharyngeal examination demonstrated a large superficial ulcer, approximately 4 × 4 cm, on the left anterior tonsillar pillar with surrounding erythema and mild exudate, without apparent deep tissue involvement. There was no clinically appreciable cervical lymphadenopathy, and there was no ocular inflammation.

Initial laboratory testing demonstrated severe neutropenia (absolute neutrophil count 0.27 × 10⁹/L), hemoglobin 112 g/L, and platelet count 143 × 10⁹/L. Automated differential reported 2.0% abnormal cells. Given the non-healing ulcer and concern for herpetic etiology versus neutropenic ulceration or underlying malignancy, she was started empirically on intravenous acyclovir (5 mg/kg per protocol) and admitted for further evaluation. As the ulcer had persisted for more than two weeks and had not improved despite prior antiviral therapy, tissue biopsy was considered as part of the standard diagnostic workup, but it was deferred because of severe neutropenia and later febrile neutropenia, and subsequently declined by the patient’s family when re-discussed.

During hospitalization, serial complete blood counts demonstrated progressive cytopenias with intermittent reports of abnormal or atypical cells on automated differentials (Table 1). Inflammatory and biochemical markers showed markedly elevated C-reactive protein, mildly elevated lactate dehydrogenase, ferritin peaking at 301.14 ng/mL, and hyperuricemia detected on follow-up (Table 2). The ferritin level was only mildly elevated and was interpreted as most consistent with an acute-phase response in the context of inflammation and infection risk rather than a hyperinflammatory syndrome such as hemophagocytic lymphohistiocytosis.

**Table 1:** Hematology trend demonstrating progressive cytopenias WBC, white blood cell count; Hb, hemoglobin; ANC, absolute neutrophil count; abnormal cells, percentage of atypical or suspicious cells reported on automated peripheral blood differential.

Day	WBC (×10⁹/L) (reference range, 4.0–11.0)	Hb (g/L) (reference range, 120–160)	Platelets (×10⁹/L) (reference range, 150–400)	ANC (×10⁹/L) (reference range, 1.5–7.5)	Abnormal cells (%)	Notes
Baseline (5 months prior)	4.7	128	314	1.26	7.0	Baseline mild neutropenia; eosinophilia noted (absolute eosinophil count: 0.47 × 10⁹/L)
Outpatient (3 months prior)	3.3	126	195	1.06	Not available	Progressive neutropenia
Outpatient (2.5 months prior)	2.95	130	235	0.66	Not available	Worsening neutropenia
Day 1 (Presentation)	4.6	112	143	0.27	2.0	Admission for persistent oral ulceration
Day 2	3.1	116	133	0.39	Not available	Severe neutropenia persists
Day 4	2.7	98	117	0.30	Not available	Pancytopenia evolving
Day 5	2.8	98	116	0.20	Not available	ANC nadir during admission
Day 6	4.8	104	73	0.33	Not available	Thrombocytopenia worsened
Day 8	3.8	100	127	0.84	2.0	Transient ANC improvement
Day 10	3.9	99	117	0.20	1.0	Abnormal cells intermittently reported
Day 13	4.0	97	101	0.52	6.0	Febrile episodes documented
Day 16	3.3	100	99	0.43	9.0	Discharged after clinical improvement
Day 29	4.3	97	100	0.17	7.0	Hematology clinic; concern for clonal process
Day 35	3.4	91	94	0.14	Not available	Pre-bone marrow evaluation
Day 36	2.9	80	76	0.11	Not available	Progressive pancytopenia
Day 39	2.6	98	67	0.14	Not available	Ongoing cytopenias

**Table 2:** Selected inflammatory markers and chemistry CRP, C-reactive protein; LDH, lactate dehydrogenase

Test	Peak value	Reference range	Day of peak	Notes
CRP (mg/L)	157.2	<5	Day 9	Markedly elevated; peaked during hospitalization
Ferritin (ng/mL)	301.14	20–300	Day 10	Upper-normal to mildly elevated; most consistent with acute-phase response in this clinical context
LDH (IU/L)	241	140–280	Day 2	Mildly elevated
Uric acid (µmol/L)	575	140–360	Day 37	Elevated; hyperuricemia detected on follow-up

Review of prior outpatient complete blood counts demonstrated mild neutropenia five months before admission (absolute neutrophil count (ANC) 1.26 × 10⁹/L) with a progressively declining ANC on subsequent tests. A definitive hematologic workup was not completed at that time because follow-up investigations were not pursued, limiting retrospective characterization of whether this represented an indolent pre-leukemic phase versus other causes of chronic neutropenia.

Autoimmune and infectious screening during admission included negative antinuclear antibody by indirect immunofluorescence (pattern AC-0), negative anti-cyclic citrullinated peptide antibody, negative anti-double-stranded DNA antibody, and negative anti-neutrophil cytoplasmic antibodies (anti-proteinase 3 and anti-myeloperoxidase). HIV antigen/antibody screening was negative. Cytomegalovirus (CMV) and Epstein-Barr virus (EBV) serology were consistent with prior exposure without evidence of acute infection (CMV IgG positive/IgM negative; EBV VCA IgG positive/IgM negative with Epstein-Barr virus nuclear antigen (EBNA) positive). Throat swab testing was documented as negative. Complement C3 and C4 were within normal limits (1.73 g/L and 0.35 g/L, respectively).

On Day 10 of admission, she developed high-grade fever (peak temperature 39.0°C) with rigors, consistent with febrile neutropenia. Empiric broad-spectrum therapy with piperacillin-tazobactam was initiated immediately. A planned ulcer biopsy was deferred during the febrile neutropenic period because the patient required urgent stabilization and treatment of febrile neutropenia, and the biopsy location at the tonsillar pillar would have required procedural support with potential bleeding and infectious risks in the setting of profound neutropenia and evolving cytopenias. In addition, the ulcer appeared superficial without a black eschar or rapidly progressive necrosis, and there were no sinonasal, facial, or orbital features to suggest an invasive fungal process at that time; therefore, close otolaryngology monitoring with reassessment for biopsy after clinical stabilization was planned. When the biopsy was re-discussed later, it was declined by the patient’s family.

The patient improved clinically over subsequent days, with resolution of fever. Intravenous ketorolac and topical lidocaine 2% mouthwash provided significant pain relief and improved oral intake. Granulocyte colony-stimulating factor was not administered because she improved with antibiotics and supportive care. She was discharged afebrile on Day 16 with an ANC of 0.43 × 10⁹/L, hemoglobin 100 g/L, and platelets 99 × 10⁹/L. Close follow-up with otolaryngology and hematology was arranged.

At hematology review on Day 29, progressive pancytopenia with persistently elevated abnormal cells raised concern for an underlying clonal hematologic process. Initial peripheral blood flow cytometry (Day 7) was non-diagnostic, showing granulocytopenia with relative monocytosis and polytypic lymphoid populations without a clear clonal expansion; however, a hematolymphoid neoplasm could not be excluded. Repeat flow cytometry (Day 33) demonstrated circulating myeloid blasts with an abnormal myeloid immunophenotype, highly suspicious for a high-grade myeloid neoplasm, including AML. She was readmitted on Day 36, and bone marrow aspirate/biopsy revealed markedly hypercellular marrow with >70% blasts and occasional Auer rods. Flow cytometry confirmed AML with monocytic differentiation. The repeat peripheral blood flow cytometry identified circulating myeloid-lineage blasts (7.7%) with an abnormal immunophenotype (myeloperoxidase (MPO) positive, CD45 dim, CD33 positive, CD38 positive, CD117 positive, CD123 positive; CD34 negative). Bone marrow aspirate and flow cytometry showed a compact blast population (approximately 78%) with monocytic differentiation markers (CD33 bright, partial MPO, partial CD64, minor CD14; CD34 negative) and occasional Auer rods on aspirate smear. CD56, CD13, and CD11b expression were not reported in the available flow cytometry panels. Acute promyelocytic leukemia was considered unlikely based on morphology and immunophenotype; *PML::RARA *fusion testing and comprehensive cytogenetic, fluorescence in situ hybridization (FISH), and molecular studies (including *FLT3* and *IDH1/2*) were sent for definitive subclassification and risk stratification.

Contrast-enhanced CT of the neck, chest, abdomen, and pelvis showed no definite extramedullary leukemic involvement. An indeterminate segment IVB liver lesion was scheduled for further characterization with contrast-enhanced MRI, but this did not alter the immediate plan. Overall, findings established AML with monocytic differentiation presenting initially as a neutropenic tonsillar ulcer.

At initial presentation, the differential diagnosis included herpetic ulceration, neutropenic ulceration, oral squamous cell carcinoma or other malignancy, viral infections (including HIV, CMV, and EBV), and autoimmune/inflammatory conditions such as Behçet disease or inflammatory bowel disease-associated aphthous ulceration. The persistence of a large non-healing oropharyngeal ulcer in the setting of progressive neutropenia supported neutropenic ulceration as the most likely proximate etiology, with an autoimmune and infectious workup non-diagnostic.

Following confirmation of AML with monocytic differentiation (Day 36), a multidisciplinary plan was made to initiate azacitidine plus venetoclax. Fertility preservation counseling was offered and accepted prior to cytotoxic therapy. At the time of treatment planning (Day 39), molecular studies (including *FLT3*, *IDH1/2*, and *PML::RARA*) were pending, with a comprehensive next-generation sequencing panel planned. The patient remained clinically stable but pancytopenic (WBC 2.6 × 10⁹/L, hemoglobin 98 g/L, platelets 67 × 10⁹/L, absolute neutrophil count 0.14 × 10⁹/L), afebrile, and was awaiting therapy initiation with close hematology-oncology follow-up.

## Discussion

This case demonstrates how recurrent oral ulceration progressing to a persistent non-healing neutropenic ulcer can represent the first clinical manifestation of AML in a young adult. Recurrent oral ulceration is common in the general population, and the majority of cases are attributed to benign recurrent aphthous stomatitis, particularly in otherwise healthy young adults [1,3]. The high prevalence of benign recurrent aphthous stomatitis in this age group may contribute to diagnostic anchoring and delay consideration of serious underlying systemic disease when atypical features are present [1,4]. In this patient, recurrent oral ulcers occurring months before presentation were initially managed as presumed herpetic ulceration with cauterization and antiviral therapy, despite progressive neutropenia documented on serial blood counts.

The differential diagnosis of recurrent and persistent oral ulceration is broad and must include hematologic disorders alongside more common benign etiologies [2,4]. While recurrent aphthous stomatitis remains the most frequent cause of recurrent oral ulcers, systematic reviews have demonstrated significant associations between hematologic deficiencies and recurrent oral ulceration, with iron, folate, and vitamin B12 deficiency being particularly relevant [1,6]. The present case underscores the critical importance of integrating clinical findings with basic hematologic screening in patients presenting with recurrent or persistent oral ulcers [4,10]. Expert consensus guidelines recommend that oral ulceration persisting beyond two weeks, failing to respond to empiric treatment, or occurring in the context of systemic symptoms or laboratory abnormalities should prompt comprehensive evaluation, including complete blood count with differential, hematinic assays, and consideration of tissue biopsy [4,10]. In this patient, severe neutropenia was documented at initial presentation, yet the oral ulcer was initially attributed to viral infection rather than prompting immediate hematologic evaluation and bone marrow assessment.

Neutropenic ulceration represents a distinct clinical entity that warrants particular attention in the differential diagnosis of oral ulceration in patients with documented neutropenia. Neutropenic ulcers are defined as mucosal ulcerations occurring in the setting of primary or secondary neutropenia, where neutropenia is considered the principal causative factor [8]. The pathophysiologic mechanism underlying neutropenic ulceration involves disruption of oral mucosal barrier homeostasis due to decreased oral neutrophil counts, impaired neutrophil extravasation from blood vessels into oral tissues, and defective reactive oxygen species production [8,9]. Neutrophils play an essential role in maintaining oral mucosal integrity through continuous recruitment to the gingival crevice and oral cavity, and quantitative or qualitative neutrophil defects predispose patients to recurrent oral ulceration, severe periodontal disease, and impaired wound healing [9]. Clinically, neutropenic ulcers are characteristically deep with irregular borders, often demonstrate necrotic or yellow-grey pseudofibrinous bases, and may paradoxically be less painful than expected, particularly in patients receiving concurrent myelosuppressive chemotherapy [8,13]. In the present case, the large tonsillar ulcer with erythematous, inflamed mucosa and mild discharge developed and persisted in the context of severe neutropenia with absolute neutrophil counts ranging from 0.11 to 0.43 × 10⁹/L, consistent with a neutropenia-driven mechanism.

Critically, the diagnosis of neutropenic ulceration should not be viewed as a terminal diagnosis but rather as a clinical syndrome that mandates systematic evaluation for the underlying cause of neutropenia [8]. In oncology practice, neutropenic ulcers may arise in patients receiving myelosuppressive chemotherapy, but primary hematologic disorders, including acute leukemia, aplastic anemia, and congenital neutropenia syndromes, must be excluded when neutropenia is unexplained or precedes cytotoxic therapy [8,13]. In this patient, neutropenia was documented months before any cancer-directed treatment and was progressive over time, which should have raised suspicion for a primary bone marrow disorder. The infectious and autoimmune work-up during the initial admission, including negative viral serology for herpes simplex virus, CMV, EBV, and HIV, as well as negative autoimmune markers, effectively excluded common secondary causes of neutropenic ulceration and should have heightened concern for underlying hematologic malignancy [4,10].

Oral manifestations are well recognized as early or even presenting features of AML and may occur in up to 90% of patients during the disease course [7,14]. These manifestations include gingival enlargement or hyperplasia, spontaneous gingival bleeding, mucosal ulceration, petechiae, mucosal pallor, and oral infections [7,14]. Gingival hyperplasia is particularly associated with monocytic and myelomonocytic subtypes of AML, occurring in approximately two-thirds of patients with acute monocytic leukemia (French-American-British classification M5) [7,15]. The pathophysiologic mechanisms are multifactorial, resulting from direct leukemic infiltration of oral tissues, profound neutropenia, thrombocytopenia, and anemia [7,14]. Multiple case reports and series document scenarios in which oral lesions were the sole or dominant initial manifestation of AML, sometimes leading to diagnostic delays when patients consulted multiple clinicians for isolated oral symptoms [7,15]. Compared with many reported presentations that emphasize gingival enlargement, bleeding, or diffuse mucosal involvement, particularly in monocytic disease, the dominant manifestation in our patient was a large neutropenic ulcer localized to the tonsillar pillar with minimal systemic symptoms at initial presentation [7,14,15]. This case also highlights two practical contributors to diagnostic delay that are important for clinicians managing persistent oral ulcers: a documented period of progressive neutropenia preceding presentation and an initially non-diagnostic peripheral blood flow cytometry that became diagnostic only on repeat testing, supporting the need for serial hematologic reassessment when cytopenias persist, and clinical suspicion remains high [4,5,10]. The present case aligns with this pattern, as the patient's only prominent symptom for approximately one month was severe throat pain with a large tonsillar ulcer, and systemic symptoms such as fever only developed during hospitalization in the context of febrile neutropenia.

Recognition of oral manifestations as potential indicators of underlying hematologic malignancy is critical for early diagnosis, particularly in young adults in whom acute leukemia may be considered less likely [7,15]. AML is the most common acute leukemia in adults, with an annual incidence of approximately 3-4.3 per 100,000 persons and a median age at diagnosis of 65-69 years [11,12]. Although the disease predominantly affects older adults, adolescents and young adults represent a distinct subgroup with different biological features and potentially better outcomes with appropriate treatment [11,12]. In young adults presenting with new-onset recurrent oral ulceration, particularly when accompanied by cytopenias, constitutional symptoms, or failure to respond to conventional therapy, maintaining a high index of suspicion for hematologic malignancy is essential [4,7]. The present case emphasizes that diagnostic algorithms for persistent oral ulcers must include age-appropriate consideration of serious underlying disease, even in otherwise healthy young adults [4].

An additional diagnostic challenge in this case was the initially non-diagnostic peripheral blood flow cytometry performed during the first admission. Clinically, this was most plausibly explained by a low circulating blast burden early in the disease course, with leukemic involvement predominantly marrow-based at that stage, so the peripheral blood sample demonstrated granulocytopenia with relative monocytosis without a definitive abnormal blast population. In the setting of profound neutropenia and low leukocyte counts, peripheral blood flow cytometry has reduced sensitivity because it depends on the presence and detectability of circulating blasts. Therefore, a negative peripheral flow cytometry result should not be considered reassuring when clinical suspicion remains high or cytopenias are persistent or progressive, and bone marrow aspirate and biopsy with morphologic assessment and flow cytometry remains the diagnostic gold standard. This case supports serial hematologic monitoring with early escalation to repeat peripheral studies and prompt bone marrow evaluation when unexplained cytopenias and refractory oral lesions persist despite initially non-diagnostic testing [4,5,10,11].

This case has several important implications for clinical practice. First, clinicians evaluating patients with recurrent or persistent oral ulceration should obtain baseline hematologic screening, including complete blood count with manual differential as part of the initial evaluation, particularly when ulcers are severe, atypical in location or morphology, or fail to respond to conventional therapy [4,6,10]. Second, the presence of neutropenia in a patient with oral ulceration should prompt systematic evaluation for the underlying cause of neutropenia, including consideration of primary bone marrow disorders when secondary causes are excluded [8,9]. Third, in patients with unexplained persistent neutropenia and oral lesions, a single non-diagnostic flow cytometry or bone marrow study should not be considered definitive, and serial evaluation guided by clinical trajectory and evolving laboratory abnormalities is warranted [4,5,11]. Fourth, interprofessional collaboration among dentistry, oral medicine, otolaryngology, and hematology is essential to ensure timely recognition of oral warning signs and appropriate diagnostic escalation [7,15]. Finally, structured diagnostic pathways for difficult oral ulcers, as outlined in expert consensus guidelines, should be implemented to reduce diagnostic delay and improve outcomes in patients with occult hematologic disease [4,10].

Limitations of this case report include the lack of histopathologic confirmation of the tonsillar ulcer, as biopsy was appropriately deferred during the febrile neutropenic period for safety reasons. Accordingly, invasive fungal infection cannot be definitively excluded histologically, although the clinical appearance and associated features were not suggestive of an invasive fungal process during the initial admission. Additionally, as with any single case report, generalizability is limited, and causality cannot be definitively established. A clinical photograph of the tonsillar/oropharyngeal ulcer was unavailable because consent for clinical photography was not obtained. In addition, representative diagnostic images (e.g., flow cytometry plots or bone marrow micrographs) could not be provided because our institutional system displays only finalized reports/results without access to image files. Nevertheless, this case contributes to the literature by illustrating the clinical presentation and diagnostic challenges of neutropenic ulceration as the initial manifestation of AML in a young woman, emphasizing the importance of systematic hematologic evaluation in patients with persistent oral ulcers and cytopenias [7,8,15].

## Conclusions

Oral manifestations, particularly neutropenic ulceration, can be the initial clinical presentation of AML in young adults and should not be dismissed as benign recurrent aphthous disease without appropriate evaluation. This case demonstrates how recurrent oral ulcers progressing to a persistent, non-healing tonsillar ulcer can obscure an underlying hematologic malignancy. While RAS remains the most common cause of recurrent oral ulcers, progressive neutropenia, refractory ulceration, or atypical features should prompt comprehensive hematologic assessment and specialist referral. Neutropenic ulceration should be recognized as a clinical syndrome requiring evaluation for underlying causes, including primary bone marrow disorders and acute leukemia. Implementing structured diagnostic pathways for persistent oral ulcers and strengthening interprofessional collaboration may reduce diagnostic delay and improve outcomes.
